# Household Sanitation and Crowding Status in Addis Health and Demographic Surveillance System (Addis-HDSS) in Addis Ababa, Ethiopia

**DOI:** 10.4314/ejhs.v34i2.3S

**Published:** 2024-12

**Authors:** Walelegn W Yallew, Nebiyou Fasil, Semira Abdelmenan, Hanna Y Berhane, Sitota Tsegaye, Dongqing Wang, Wafaie Fawzi, Meaza Demissie, Alemayehu Worku, Yemane Birhane

**Affiliations:** 1 Department of Global Health and Health Policy, Addis Continental Institute of Public Health; 2 Department of Epidemiology and Biostatistics, Addis Continental Institute of Public Health; 3 Department of Nutrition and Behavioral Science, Addis Continental Institute of Public Health; 4 Department of Global and Community Health, College of Public Health, George Mason University, Fairfax, Virginia, United States of America; 5 Department of Global Health and Population, Harvard T.H. Chan School of Public Health, Harvard University, Boston, Massachusetts, United States of America

**Keywords:** Sanitation, Household crowding index, Ethiopia

## Abstract

**Background:**

Access to sanitation and healthy housing conditions are essential for public health, reducing the spread of diseases and improving overall well-being. However, millions of people, particularly in low-income countries, still lack access to basic sanitation and housing facilities. This study assessed household sanitation and crowding status in a rapidly developing urban area of Addis Ababa.

**Methods:**

Data were extracted from the household census conducted from December 2022 to January 2023 at the Addis-HDSS site. Availability of basic sanitation facilities was defined as the presence of privately owned sanitation facilities within the household. Household crowding was measured by the number of occupants per bedroom. Multivariable logistic regression was used to identify factors associated with access to sanitation facilities (STATA/SE 14.2). A p-value of <0.05 was considered statistically significant.

**Results:**

The study included 30,533 households. Overall, 76.37% (95% CI: 74.86–77.2) lacked access to basic sanitation facilities. Most households (67.42%) lived in overcrowded housing. Educational status of the household head and household size were significantly associated with sanitation access. Households with college-educated heads were more likely to have access to basic sanitation (AOR 2.52, 95% CI: 2.27–2.79), while overcrowded households were less likely to have such access (AOR 0.06, 95% CI: 0.040–0.063).

**Conclusions:**

A large proportion of households lacked basic sanitation facilities and lived in overcrowded housing, which increases the risk of infectious disease transmission. Improving sanitation and housing conditions is crucial for reducing health risks and improving public health outcomes.

## Introduction

Despite global efforts, a significant portion of the population still lacks access to basic sanitation facilities(1). According to the World Health Organization, in 2022, only 2.4 billion people worldwide had access to safe sanitation (1, 2). Ethiopia has one of the lowest access rates, with only 20% of households using improved toilet facilities (3). In urban areas, overcrowding is also a major public health concern, as it exacerbates the spread of infectious diseases, including diarrheal diseases, tuberculosis, and respiratory infections (4-6). Sanitation interventions have been shown to increase both the availability and use of latrines (7). Understanding the factors influencing sanitation access and household overcrowding is critical for developing effective public health policies. Previous studies have identified factors associated with access to sanitation, including household wealth, education status, and crowding (8–12). However, few large-scale studies have been conducted in Addis Ababa.

This study aims to investigate the proportion of households with access to basic sanitation and the prevalence of overcrowding. It also asses to identify the socioeconomic factors associated with sanitation access.

## Methods

**Figure 1:**
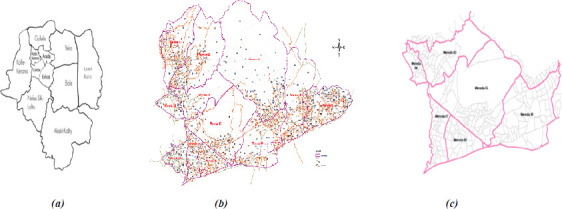
Map of the sub-cities of Addis Ababa (a), Woredas of Yeka sub-city (b), and Addis-HDSS site(c)

**Study setting**: The Addis Health and Demographic Surveillance System (Addis-HDSS) is located in Yeka sub-city, Addis Ababa, the capital city of Ethiopia. Addis Ababa has a population of approximately 4 million people, with a population density of 5,165 individuals per square kilometer (13). The city is growing rapidly, with an annual growth rate of 4.4%, making it one of the fastest-growing cities in the world(*Figure 1*).

**Study design and population**: Data were obtained from the Addis-HDSS census conducted from December 2022 to January 2023. All households in the Addis-HDSS were eligible for the study, with heads of households or any adult household members serving as respondents.

**Data collection**: A structured questionnaire, adapted from a similar system used in Ethiopia, was used to collect data on housing and household member characteristics(14). Data were collected electronically using tablets. Fieldworkers were trained on data collection methods, and quality checks were performed during the fieldwork to ensure accuracy.

### Measurement

**Sanitation availability**: Defined as the presence of privately owned sanitation facilities (e.g., flush toilets, pit latrines, VIP latrines)(15).

**Crowding status**: Assessed using a crowding score, which was calculated by dividing the number of household members by the number of bedrooms. Households were categorized as “Not Crowded” (score ≤ 1), “Crowded” (score > 1), or “Overcrowded” (score ≥ 3) (16).

**Data management and analysis**: Data were managed using STATA/SE 14.2. Descriptive statistics were used to assess sanitation and crowding status, and logistic regression was used to identify factors associated with sanitation access. Results are presented as odds ratios (OR) with 95% confidence intervals (CI).

**Ethical considerations**: Ethical approval was obtained from the Addis Continental Institute of Public Health and the Addis Ababa Health Bureau. Written informed consent was obtained from all participants.

## Results

The study included 30,533 households. The median household size was 3, with an interquartile range of 3. A total of 29.04% of households owned their homes, and the majority of household heads were married (56.4%), completed secondary education (29.4%), and were employed (26.4%).

**Figure 2:**
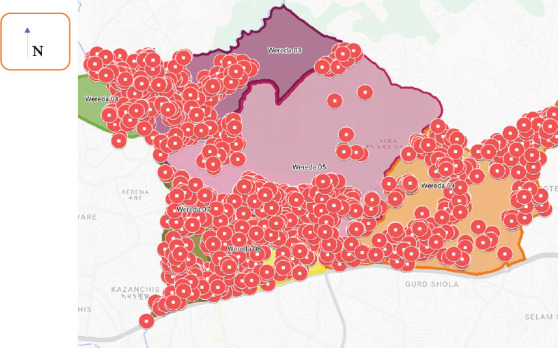
Map of the sub-cities of sanitation status in Addis -HDSS sites in 2022

**Sanitation access**: Only 23.26% (95% CI: 22.8–23.7) of households had access to basic sanitation facilities(Table 2, Figure 2).

**Table 2:** Households access to basic sanitation facility and household crowding in Addis HDSS, Addis Ababa, Ethiopia

Variable		Frequency	Percent	95% CI
Households' sanitation status	Had no basic sanitation	23431	76.3	74.86, 77.2
	Had basic Sanitation	7102	23.26	22.8, 23.7
Household crowding	Not crowded	3689	12.08	11.7- 12.5
	Crowded	20584	67.42	66.9, 67.9
	Overcrowded	6260	20.50	20.01,21.0

**Figure 3:**
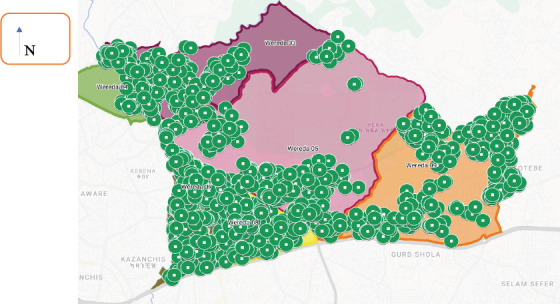
Map of the sub-cities of Household crowded status in Addis-HDSS sites in 2022

**Crowding status**: 12.08% of households were not overcrowded, while 67.42% were crowded, and 20.50% were considered overcrowded(Table 2 & *Figure 3*).

**Logistic regression**: After controlling for potential confounders, households with college-educated heads were more likely to have access to sanitation (AOR 2.52, 95% CI: 2.27–2.79). In contrast, overcrowded household facilities had significantly lower odds of accessing sanitation facilities (AOR 0.06, 95% CI: 0.040–0.063)(Table 3).

**Table 3:** Factors associated with access to sanitation facilities of households in Addis HDSS, Addis Ababa, 2022

Characteristics	Access to basic Sanitation facilities		Crude OR	Adjusted OR

	Yes	No		
**Household Size**				
**Mean (±SD)**	3.98(1.89)	3.37(1.67)	1.21(1.19,1.23)***	1.59(1.566,1.63)***
**Median (IQR)**	4(2)	3(2)		
**Education status of Head of the Household**				
**No formal** **education/Preschool**	628	3247	1	1
**Primary(1-8)**	1058	6598	0.83(0.75,0.92)***	0.80(0.72,0.90)**
**Secondary(9-12)**	1791	7001	1.32(1.19,1.46)***	1.21(1.09,1.35)**
**Vocational/Technical**	418	1206	1.79(1.56,2.06)***	1.53(1.34,1.78)
**College/University**	3053	4960	3.18(2.89,3.51)***	2.52(2.27,2.79)***
**Household crowding status**				
**Not crowded**	1768	1921	1	1
**Crowded**	4223	16361	0.28(0.26,0.30)***	0.17(0.16,0.183)***
**Overcrowded**	1111	5149	0.23(0.21,0.26)***	0.06(0.040.063)***

**NB: p-value** - * p-value (0.01, 05), ** p-value (0.001, 0.01), *** p-value (<0.001)

## Discussion

Only about 25% of households had access to basic sanitation, and just 1 in 10 lived in non-crowded housing. Household size, educational status of the head of the household, and crowding were significantly associated with sanitation access.

Low access to sanitation increases the risk of infectious diseases such as diarrhea and cholera (17–21). Studies in Ethiopia have also shown low levels of sanitation access, contributing to contamination of water sources and poor health outcomes (22-24).

Crowding is a significant public health concern, as it promotes the spread of respiratory infections and other diseases due to inadequate ventilation and close contact (25, 26). Overcrowded households face challenges in maintaining sanitation, as space limitations hinder the construction of adequate facilities (12, 16). Large families are particularly at risk, as they require more space and resources to meet their needs (11).

The findings highlight the need for urgent interventions to improve sanitation access and reduce overcrowding. Addressing these issues will help mitigate the spread of infectious diseases, including emerging threats like COVID-19.

This study relied on self-reported data and did not assess the functional status of sanitation facilities. Additionally, the exact size of housing units was not measured. Future studies should include more objective measurements of these factors.

In conclusion, three-quarters of households in the study area lacked access to basic sanitation, and nearly 90% lived in overcrowded housing. Poor sanitation and overcrowding were more prevalent in households with uneducated heads and larger family sizes. Improving sanitation and housing conditions is essential to prevent the spread of infectious diseases in rapidly growing urban areas.

## Figures and Tables

**Table 1:** Socio-demographic characteristics of household and household head in Addis HDSS, Addis Ababa, 2022

Characteristics	Frequency	Percent
**Household ownership**		
**Own**	8867	29.04
**Rented from the government**	6345	20.78
**Rented from Individuals**	12180	39.89
**Cohabited (no payment)**	2464	8.07
**Other (Military housing, Church)**	677	2.22
**Household Size**		
**Mean(+SD)**	3.51(1.75)	
**Median (IQR)**	3(3)	
**Household head marital status**		
**Single (never married)**	4827	16.11
**Married (monogamous)**	16882	56.35
**Divorced**	2049	6.84
**Widow/Widower**	5090	16.99
**Separated**	1112	3.71
**Household head educational status**		
**No formal education/Preschool**	3875	12.93
**Primary (1-8)**	7656	25.55
**Secondary (9-12)**	8792	29.35
**Vocational/Technical**	1624	5.42
**College/University**	8013	26.75
**Household head occupational status**		
**Student**	160	0.53
**Not employed**	5334	17.80
**Occasionally employed**	1246	4.16
**Seasonal employment**	685	2.29
**Temporary employed**	4529	15.12
**Permanently employed**	7894	26.35
**Self-employed**	7034	23.48
**Retired**	3081	10.28

## References

[1] United Nations (2023). Human Rights Council. Resolution Adopted by the Human Rights Council. 18/1. The Human Right to Safe Drinking Water and Sanitation [Internet].

[2] United Nations Children's Fund (UNICEF), World Health Organization (WHO) (2023). Progress on household drinking water, sanitation and hygiene 2000–2022: special focus on gender.

[3] Ephi EPHI, FMoH FM of H, ICF (2021). Ethiopia Mini Demographic and Health Survey 2019.

[4] Hosseini LJ, Samadi AH, Woldemichael A, Gharebelagh MN, Rezaei S, Rad EH (2021). Household Overcrowding in Iran, a Low-middle-income Country: How Major of a Public Health Concern Is It?. J Prev Med Public Health.

[5] Wolf J, Hubbard S, Brauer M, Ambelu A, Arnold BF, Bain R (2022). Effectiveness of interventions to improve drinking water, sanitation, and handwashing with soap on risk of diarrhoeal disease in children in low-income and middle-income settings: a systematic review and metaanalysis. Lancet.

[6] Kamis C, Stolte A, West JS, Fishman SH, Brown T, Brown T (2021). Overcrowding and COVID-19 mortality across U.S. counties: Are disparities growing over time?. SSM Popul Health.

[7] Garn JV, Sclar GD, Freeman MC, Penakalapati G, Alexander KT, Brooks P (2017). The impact of sanitation interventions on latrine coverage and latrine use: A systematic review and metaanalysis. Int J Hyg Environ Health.

[8] Afework A, Beyene H, Ermias A, Tamene A (2022). Moving Up the Sanitation Ladder: A Study of the Coverage and Utilization of Improved Sanitation Facilities and Associated Factors Among Households in Southern Ethiopia. Environ Health Insights.

[9] Gaffan N, Kpozèhouen A, Dégbey C, Glèlè Ahanhanzo Y, Glèlè Kakaï R, Salamon R (2022). Household access to basic drinking water, sanitation and hygiene facilities: secondary analysis of data from the demographic and health survey V, 2017–2018. BMC Public Health.

[10] Muslim EU, Stanikzai MH, Wasiq AW, Khan A, Sayam H (2021). The Availability of Improved Sanitation Facilities and Its Associated Factors in the 12th District of Kandahar City, Afghanistan. J Environ Public Health.

[11] Asnake D, Adane M (2020). Household latrine utilization and associated factors in semi-urban areas of northeastern Ethiopia. PLoS One.

[12] Lapidot Y, Reshef L, Maya M, Cohen D, Gophna U, Muhsen K (2022). Socioeconomic disparities and household crowding in association with the fecal microbiome of school-age children. NPJ Biofilms Microbiomes.

[13] Ethiopian Stastical service (2023). Ethiopian Stastical service 2023 Popullation Projection [Internet].

[14] CSA/Ethiopia CSA, ICF (2017). Ethiopia Demographic and Health Survey 2016.

[15] Ethiopian Public Health Institute, Federal ministry of health (Ethiopia), ICF (2020). Ethiopia Mini Demographic and Health Survey 2019.

[16] WHO (2018). WHO Housing and Health Guidelines. WHO Housing and Health Guidelines [Internet].

[17] Wagari S, Girma H, Geremew A (2022). Water, Sanitation, and Hygiene Service Ladders and Childhood Diarrhea in Haramaya Demographic and Health Surveillance Site, Eastern Ethiopia. Environ Health Insights.

[18] Mulatu G, Ayana GM, Girma H, Mulugeta Y, Daraje G, Geremew A (2022). Association of drinking water and environmental sanitation with diarrhea among under-five children: Evidence from Kersa demographic and health surveillance site, eastern Ethiopia. Front Public Health.

[19] Workie GY, Akalu TY, Baraki AG (2019). Environmental factors affecting childhood diarrheal disease among under-five children in Jamma district, South Wello zone, Northeast Ethiopia. BMC Infect Dis.

[20] Challa JM, Getachew T, Debella A, Merid M, Atnafe G, Eyeberu A (2022). Inadequate Hand Washing, Lack of Clean Drinking Water and Latrines as Major Determinants of Cholera Outbreak in Somali Region, Ethiopia in 2019. Front Public Health.

[21] Mara D, Lane J, Scott B, Trouba D (2010). Sanitation and Health. PLoS Med.

[22] Novotný J, Mamo BG (2022). Household-level sanitation in Ethiopia and its influencing factors: a systematic review. BMC Public Health.

[23] Okullo JO, Moturi WN, Ogendi GM (2017). Open Defaecation and Its Effects on the Bacteriological Quality of Drinking Water Sources in Isiolo County, Kenya. Environ Health Insights.

[24] Huda TMdN, Schmidt WP, Pickering AJ, Mahmud ZH, Islam MS, Rahman MdS (2018). A Cross-Sectional Study of the Association between Sanitation Type and Fecal Contamination of the Household Environment in Rural Bangladesh. Am J Trop Med Hyg.

[25] Pelissari DM, Diaz-Quijano FA (2017). Household crowding as a potential mediator of socioeconomic determinants of tuberculosis incidence in Brazil. PLoS One.

[26] Melki IS, Beydoun HA, Khogali M, Tamim H, Yunis KA, National Collaborative Perinatal Neonatal Network (NCPNN) (2004). Household crowding index: a correlate of socioeconomic status and inter-pregnancy spacing in an urban setting. J Epidemiol Community Health.

